# The application of growth factors in bone tissue engineering delivery systems and collaborative innovation strategies

**DOI:** 10.1080/07853890.2026.2670149

**Published:** 2026-05-18

**Authors:** Meiqi Xu, Hongjuan Cao, Lin Wu

**Affiliations:** Department of Stomatology, Shengjing Hospital of China Medical University, Shenyang, China

**Keywords:** Bone tissue engineering, growth factors, biomaterial scaffolds, bone regeneration

## Abstract

**Background:**

Although bone tissue has inherent healing ability, bone regeneration of critical size defects still requires strategic intervention. As key signalling molecules, growth factors (GFs) coordinate bone formation and angiogenesis. However, their clinical application faces several challenges, including short half-lives, off-target effects and insufficient spatiotemporal control.

**Objective:**

This article reviews the role of key GFs in bone tissue engineering (BTE) and critically evaluates advanced delivery systems based on the core translational concepts, aiming to promote safer and more effective bone repair methods.

**Content:**

Moving beyond traditional material-based classifications, this review explores how biomaterial platforms achieve precise release kinetics and spatial control of key GFs, such as bone morphogenesis proteins (BMPs) and vascular endothelial growth factor (VEGF). In addition, it also emphasizes innovative strategies, including cell-based systems, stimulus response platforms and advanced manufacturing technologies such as 3D printing and electrostatic spinning. These strategies enhance the stability and target distribution of GFs. The key is that it describes safety problems and transformation obstacles, including off-target effect, ectopic bone formation and manufacturing scalability.

**Conclusion:**

GFs continue to show great potential in the field of bone regeneration. Future progress will depend on bridging the gap between intelligent biomaterial design and clinical application by solving key transformation obstacles related to manufacturing technology and systemic safety.

## 1. Introduction

**Figure 1.**
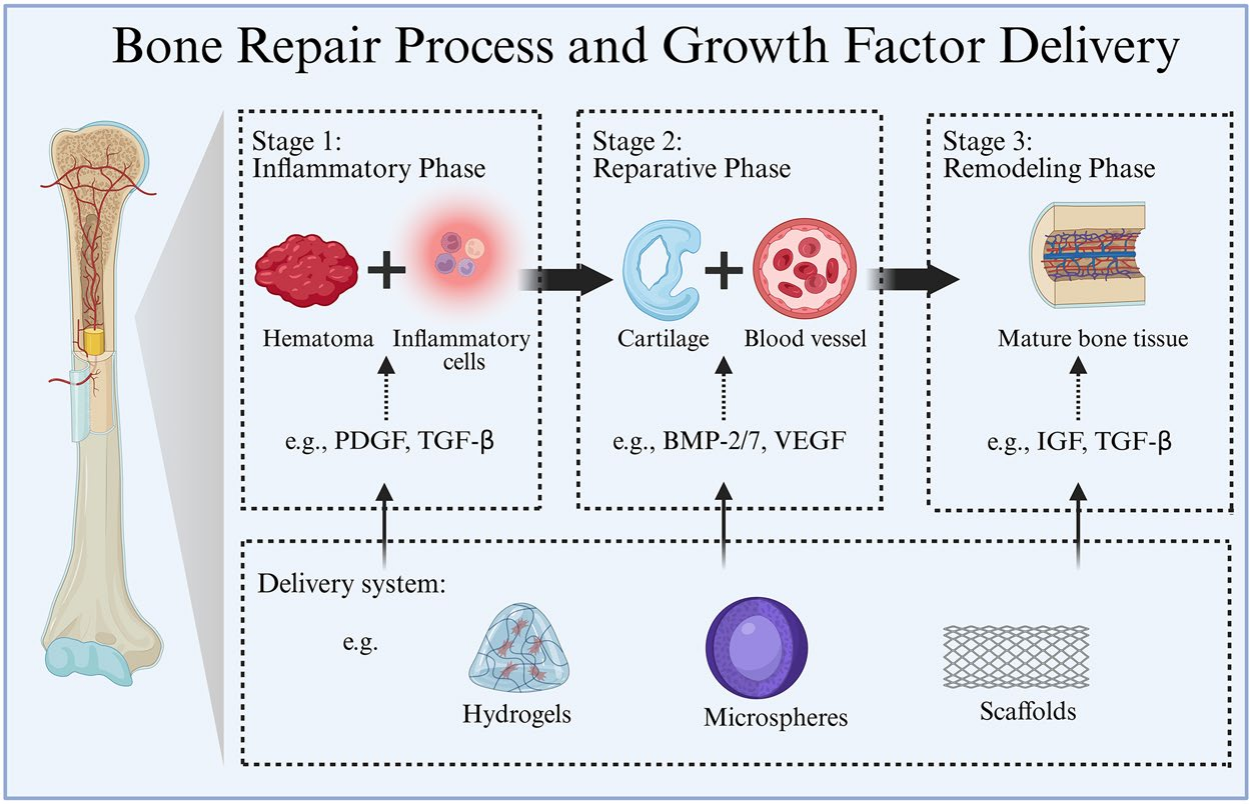
Bone repair process and GF delivery. Bone tissue engineering (BTE) mimics the natural healing cascade through three time-controlled stages: (1) Inflammatory Phase: TGF-β and PDGF recruit progenitor cells to the injury site; (2) Reparative Phase: Continuous release of VEGF and BMPs coordinates angiogenesis and ossification; (3) Remodelling Phase: Downward modulation of signalling promotes matrix mineralization and structural maturation. Precise spatiotemporal control of GF release is essential for achieving functional bone regeneration.

Bone is a highly active connective tissue that provides the body with the necessary mechanical strength and structural integrity [1]. Although most fractures can heal independently, more complex or severe bone injuries typically require external treatment for full recovery [2]. Bone healing occurs in a series of events: haematoma formation, inflammation and soft and hard callus formation. All these events are regulated by various cytokines and growth factors (GFs) [3–5] (Figure 1). Although existing bone treatments can be effective in clinical settings, they are not without challenges. First, autografts and allografts suffer from limited availability, and there are complications at the donor site, as well as risks of immune rejection or disease transmission. These limitations have hindered their broader application, necessitating innovative regenerative approaches to restore the structure and function of severely injured bone tissue [6].

GFs are key strategies for tissue regeneration by creating a osteogenic microenvironment. An effective way for restoring bone tissue structure and function is to reconstruct the microenvironment and activate the signalling pathway to promote the regeneration at the damaged site [7]. The inculcation of biomaterials with cells and GFs into the regeneration process of simulated tissues is functionally and structurally helpful [8]. GF-based treatments aim to repair damaged tissue by using the embryonic and postnatal developmental processes [9,10]. Bone regeneration is driven by a complex physiological process, which is regulated by various biochemical elements that occur in a specific order. Using GFs can promote or inhibit bone healing. GFs affect cell behaviour by binding to specific receptors on the cell membrane, thus affecting cell proliferation, self-renewal, migration and differentiation [11–13]. Soluble proteins combine with their specific receptors, such as tyrosine kinase receptors, triggering reactions that determine cell activity or fate [14]. The typical GF signalling pathway involves receptors related to the extracellular matrix (ECM), and various signalling pathways are used for bone formation. Members of the TGF-β superfamily usually use serine/threoline kinase receptors in combination with fibroblast growth factor (FGF) receptors for the formation of osteofibroblasts [15].

Predicting the effectiveness of GFs is still a complex task due to variables such as the dose, the specific location of the application, the size of the defect and the differences in animal models. When these soluble molecules are injected or applied directly to the damaged area, they affect not only the expected tissue, but also the neighbouring tissue. In order to solve non-selective problems, the most important practical way is to use the controlled release system. This technology is widely used, and bioactive substances can be gradually released through biomaterial carriers [16,17]. When the surrounding tissue does not have the ability to regenerate naturally, providing a stent alone may not ensure successful tissue repair. It is crucial to pair the scaffold with cells and/or signalling molecules that promote tissue regeneration [18]. GF delivery can be achieved by ming free GFs into the vector by physical embedding or by using covalent or non-covalent bonds. The choice of fixed technology is influenced by specific GFs and expected delivery methods [19].

A key consideration in the design of the GF delivery system is to create a carrier that provides a controlled release curve customized according to the time and space requirements of regenerative tissue [20]. In order to meet these clinical and technical needs, this review systematically reorganizes the progress of the GF delivery system around the core transformation concept: release dynamics, space control, GF stability, systemic safety and transformation disorders. By evaluating the feasibility, current controversy and safety characteristics of these advanced platforms, this article provides new insights for the development of efficient, accurate and safe clinical bone repair strategies.

## 2. Core applications of GFs in BTE

Due to their strong ability to promote bone regeneration, GFs have been widely utilized in the repair of bone tissue. Naturally, bone is rich in various GFs, including TGF-β, BMP, VEGF, insulin-like growth factor (IGF), FGF and PDGF, all of which play crucial roles in the process of osteogenesis [21–23]. Each of these GFs is involved in different stages of bone healing. The healing of bone damage is a multi-facetted process, including a series of catabolism and anabolic activities, and finally forms a new functional bone tissue. The bone repair process is divided into three stages: acute inflammation period, repair period and remodelling period [24]. Different types or sources of cells secrete various cytokines and GFs which take part in each of these phases (Table 1).

**Table 1.** Summary table of GFs functions and applications.

Growth factor	Mechanism & phase	Intervention / dose (unit)	Model type/species	Key translational findings	References
TGF-β	Dual regulation; Full course	1–50 ng/mL	MSCs; Mouse models	Dose-dependent (low-promote/high-inhibit);	[25–27]
		2.3 ng/mL	ESsT cells; CTG fibroblasts	Synergizes with IGF-1;	
		–	Human models	OA-related dysregulation.	
BMPs (BMP-2/4/6/7/9)	Osteoinduction; Repair/Remodelling	50 ng/mL	MSCs	BMP-2/7 exhibit peak potency;	[28–31]
		100 ng/scaffold	Mouse models	Sustained release optimizes bone volume/quality;	
		50 ng/mL	Rabbit models; MSCs	Synergizes with TGF-β;	
		100 ng/mL	Rat models; MSCs	BMP-6 promoted osteochondral repair and vascularization.	
VEGF	Angiogenesis; Early/Mid stage	100 ng/mL	Mouse models; MSCs	Dose-sensitive vascularization;	[32,33]
		–	Rat models	VEGF-driven calvarial repair.	
IGF	Homeostasis; Mid stage	–	–	Multi-pathway synergy beyond direct receptor activity;	[34,35]
		100 μg/kg bodyweight	Rat models	Sourced from BMSCs and platelets.	
FGF	Recruitment; Early stage	500 ng /scaffold	hADSCs; HUVECs; Mouse models	FGF-18 critical for progenitor expansion;	[36,37]
		1 μg/mL	PDLSCs; RAW264.7; Rat models	Modulates immune microenvironment.	
PDGF	Chemotaxis; Initiation phase	–	–	PDGFR-α signalling participates in skeletal development;	[38,39]
		–	–	PDGFR-β signalling promotes vascularized osteogenesis.	
PRP	Burst release; Full course	300 μL/scaffold	MC3T3-E1 cells	Synergizes with bioceramics;	[40,41]
		–	Rat models; MSCs	Composite microspheres extend GF bioactivity.	
CGF	Sustained release; Full course	–	Rabbit models	Synergizes with collagen to accelerate bone formation;	[42,43]
		100 μL/mL	Rabbit models; MSCs	Enhances osteogenesis within various composite scaffolds.	
PRF	Autologous scaffold; Full course	16.334 μg/μL	MSCs	Accelerates HMSC differentiation;	[44,45]
		4–5 mL/scaffold	Human models	High clinical success in oral/maxillofacial repair.	

*Note:* PRP, platelet-rich plasma; CGF, concentrated growth factors; PRF, platelet-rich fibrin; OA osteoarthritis; ESsT, extraction socket soft tissue; CTG, connective tissue graft; hADSCs, human adipose-derived stem cells; HUVECs, human umbilical vein endothelial cells; PDLSCs, periodontal ligament stem cells; RAW264.7, mouse monocyte-macrophage leukaemia cells; MC3T3-E1, Mouse embryo osteoblast precursor cells.

### 2.1. TGF-β superfamily: complexity of Bi-directional regulation

The members of the TGF-β gene superfamily include activins, BMPs and growth and differentiation factors (GDFs). All of these proteins are crucial to the growth and differentiation of many different cell types, including osteoblasts. Osteoblasts build TGF-β1 and TGF-β2 that get integrated into the mineralized matrix of bone. TGF-β1 [46] is a crucial cytokine required for proper bone regeneration. Furthermore, the outcome of fracture union depends on this cytokine.

The multifunctional roles of TGF-β in bone biology suggest potential therapeutic strategies dependent on the context. Physiological repair, it directs early regenerative events, such as angiogenesis and stem cell recruitment [47], and it may also function as a GF-preserving marker of bone allografts [48]. Evidence indicates that the therapeutic effects of TGF-β exhibit significant background dependence. Yu et al. [49] show that exogenous TGF-β1 can repair damaged osteogenic function by regulating the ubiquitin-proteasome pathway. However, in an osteogenesis imperfecta model, Greene et al. [50] unexpectedly increased bone mass by inhibiting TGF-β by neutralizing antibodies. This shows that in the pathological state, the TGF-β strategy should shift from simple supplementation to precise regulation.

### 2.2. BMPs: from clinical gold standard to safety challenges

BMPs belong to the TGF-β superfamily. They are known for driving ectopic bone formation and chondrogenic differentiation. BMPs are essential in BTE. These potent GFs can convert perivascular mesenchymal cells and bone marrow stromal cells into chondrocytes and osteoblasts. In recent years, research into BMPs and their potential application in bone regeneration research has shown a global surge [51–53].

BMPs, especially BMP-2 and BMP-4 [54], occupy a central position in BTE because of their excellent osteogenesis potential. However, its clinical transformation path is significantly complex. The U.S. Food and Drug Administration (FDA) first approved the recombinant human BMP-2 (rhBMP-2) product for lumbar anterior intervertebral fusion in 2002. In 2007, the U.S. FDA approved rhBMP-2 for maxillary sinus enlargement and alveolar ridge enlargement to preserve the alveolar cavity [55,56]. Long-term clinical research has further confirmed its clinical application value [57]. The combination of implants with bone grafting and periosteum whether combined with rhBMP-2 has been stable for more than 17 years.

Despite its clinical dominance, the main challenge of the INFUSE^®^ (rhBMP-2/absorbable collagen sponge [ACS]) system is its dependence on superphysiological doses – usually 1.5 mg/mL, which is significantly higher than endogenous levels [58]. When using ACS as a carrier, it usually leads to sudden release, of which up to 50% of BMP-2 will be lost in the first few days. This requires the initial use of high doses to ensure long-term bone-inducing effects, but at the same time, it can cause serious dose-dependent adverse reactions, including ectopic bone formation, osteolysis and life-threatening prevertebral oedema [59]. Clinical evidence further shows that uncontrolled BMP release may lead to airway oedema, nerve damage and local bone absorption caused by excessive inflammation. For example, Du et al. [60] warned that although BMP-4 showed excellent performance *in vitro*, its *in vivo* administration could cause serious structural damage.

In order to reduce the risk of high doses, research is turning to high-efficiency, low-dose delivery systems [61,62]. However, clinical transformation is hindered by the manufacturing complexity of maintaining GF biological activity in the process of large-scale production and the strict supervision of combination products [63]. The high cost of clinical trials and the lack of a standardized safety framework for long-term low-dose exposure further increase the complexity of these innovative therapies being approved compared with existing high-dose standards. The current cutting-edge strategy emphasizes synergistic administration, such as the combination of rhBMP-2 with antibacterial or anti-inflammatory agents [29], to optimize the treatment effect through precise release kinetics rather than simple physical adsorption.

### 2.3. VEGF: coupling angiogenesis and osteogenesis

**Figure 2.**
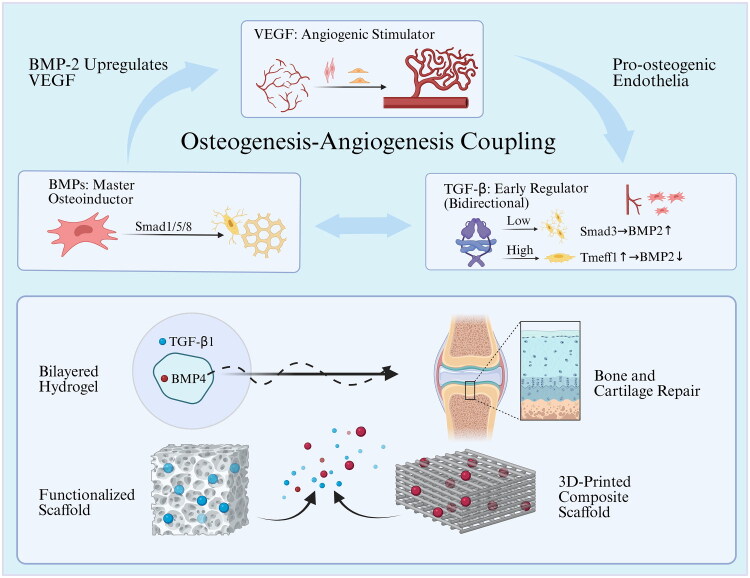
The synergistic roles of TGF-β, BMPs and VEGF in bone healing. TGF-β influences osteogenic differentiation bidirectionally, BMPs mainly drive bone development, and VEGF promotes angiogenesis; these three factors are interrelated by supporting the signal network of bone vascularization. Advanced delivery platforms, including bilayered hydrogels, functionalized scaffolds and 3D-printed composite systems, enable the precise spatiotemporal release of these bioactive factors to mimic the natural healing cascade.

VEGF serves as a potent and targeted angiogenic cytokine synthesized by various cell types, such as endothelial cells and osteoblasts, at the sites of fractures. It plays a crucial role in promoting neovascularization and the formation of new bone during both intramembranous and endochondral ossification [64,65] (Figure 2). There are seven isoforms of VEGF—namely VEGF-A, -B, -C, -D, -E, -F and placental GF-A—that are structurally akin homodimeric glycoproteins. These isoforms exhibit different binding affinities to tyrosine kinase receptors, specifically VEGFR-1, -2 and -3 [66].

The uniform distribution of VEGF protein and matrix maintains the natural relationship between angiogenesis and bone generation, providing a promising and practical method for building vascularized bones. However, the traditional direct injection method has limited effect, while modern research focusses on matrix binding. Huang et al. [67] used Dihydroxyphenylalanine coating material to simulate the natural microenvironment of allogeneic bones, which enhanced the bone formation signal, while Chen et al. [68] used Hydroxyapatite/Chitosan stents in the rabbit femur model to achieve stable VEGF release, confirming the accelerating effect of vascularization in the process of remineralization.

### 2.4. Other key GFs: metabolism and sequential regulation

#### 2.4.1. IGF

The IGFs secreted by bone cells have both immediate autosecretion and parasecretory regulatory functions. These factors are also integrated into the bone matrix and may be released during bone absorption. Among the GFs stored in the bone matrix [69], IGF-I and IGF-II are the most common. Research by Wang et al. [34] identified bone marrow mesenchymal stem cells (BMSCs) and megakaryocytes/platelets as key contributors to IGF-1 production in bone. IGF-I not only directly promotes the increase of muscle mass and bone density, but also indirectly affects the system through mechanical interaction between the musculoskeletal system. Therefore, the comprehensive effect of IGF-I far exceeds its direct receptor-mediated effect [70]. In the research of Park et al. [71] oral freeze-dried plant cells can achieve the continuous release of functional IGF-1, which can stimulate a variety of cell proliferation and osteogenic differentiation, significantly improve bone regeneration in the fracture model of diabetic mice, and improve bone density and bone volume. This oral delivery system, specifically tailored for diabetic fracture models, offers a highly attractive non-invasive translational pathway for bone defect patients suffering from systemic metabolic diseases.

#### 2.4.2. FGF

In the FGF family, FGF-2, −9 and −18 are essential for bone healing [72]. Scaffold materials incorporating FGFs can significantly enhance bone-conducting and bone-inducing properties [73]. In recent years, research has shifted focus from single carriers to precisely controlled release achieved through advanced manufacturing technologies. For instance, Zhang et al. [74] utilized emulsion electrospun polyurethane fibre membranes, which significantly enhanced cellular vascularization capability through sustained release of FGF-2. The core/shell fibre scaffold designed by Ding et al. [37] achieved sequential release of FGF-2 and BMP-2, which not only promoted osteogenic differentiation but also optimized the bone immune microenvironment by inducing M2-type macrophage polarization. These studies have jointly confirmed the key role of FGFs in improving the blood circulation and microenvironment of the periodontal bone defect model, and provide a key experimental basis for the development of high-efficiency periodontal regeneration membrane materials.

#### 2.4.3. PDGF

There are five isoforms of PDGF (AA, BB, AB, CC and DD). The secreted signalling molecules that bind PDGF receptor α or β function in a paracrine or autocrine way in physiological or pathological processes [73]. The deleterious effects resulting from loss of PDGFR-α signalling are more diverse than those of PDGFR-β [74]. In contrast, the PDGFR-β pathway is essential for the development of the vascular system and the initiation of haematopoiesis [36]. PDGF-BB is pivotal in regulating macrophage polarization and reducing bone resorption [37]. However, its effect is time-sensitive. In early osteoarthritis, excessive PDGF-BB may abnormally induce abnormal angiogenesis and accelerate disease progression [75]. In contrast, in the bone defect model, loading PDGF on the β-tricalcium phosphate (β-TCP) bracket shows a better repair promotion effect [76].

## 3. Engineering GFs stability, release kinetics and spatial control

**Figure 3.**
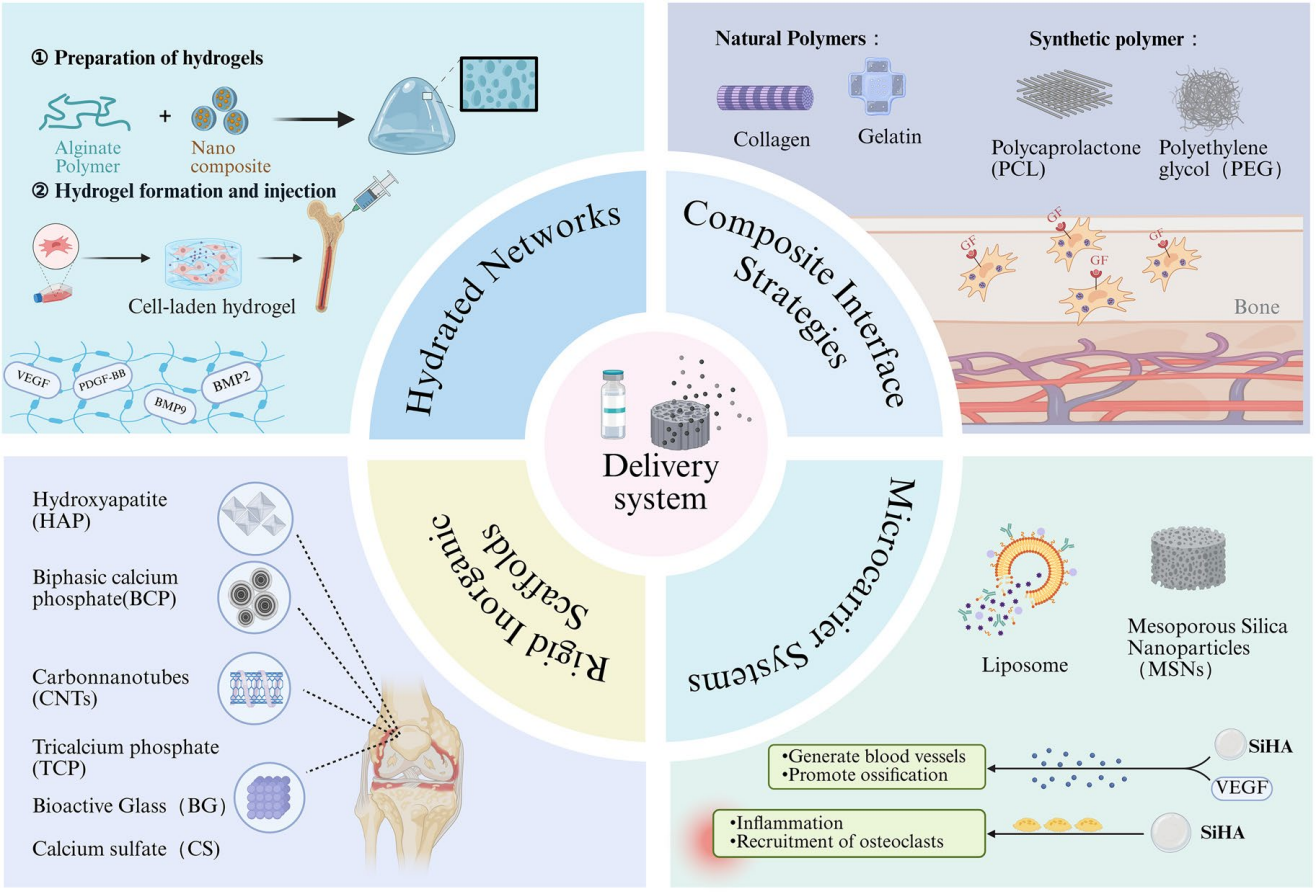
Classification and Characteristics of BTE Delivery Systems. 1. Hydrated Networks: (e.g. Alginate, Gelatine) Injectable and stimuli-responsive hydrogels for filling irregular, non-load-bearing defects; 2. Rigid Inorganic Scaffolds: (e.g. HAP, BCP, CNTs) High-strength bioceramics providing mechanical support and osteoconductivity; 3. Composite Interface Strategies: (e.g. PCL, PEG, Collagen) Hybrid polymers featuring tailorable degradation and biomimetic ECM interfaces; 4. Microcarrier Systems: (e.g. MSNs, Liposomes) Advanced vehicles for precise, sustained, or responsive release kinetics.

The key challenge faced by GF treatment is that when applied directly to the dynamic *in vivo*, it will quickly undergo enzymatic degradation and lose biological activity [77]. Thus, the primary goal of any delivery system is to regulate release kinetics while preserving GF stability. The ideal dynamic curve largely depends on the healing stage. Early inflammation and cell collection may require initial explosive release, while long-term bone formation and vascularization must rely on continuous release [78]. This section discusses how to meet these specific dynamic, spatial and stability requirements by designing different biomaterial platforms ranging from hydrated networks for non-load-bearing defects to microcarrier systems for sequential delivery (Figure 3 and Table 2).

**Table 2.** Comparison of delivery system types and characteristics.

Delivery system type	Material examples	Critical benefits	Translational barriers	Intervention/dose (Unit)	Model type/species	Application examples	References
Hydrated Networks	Gelatine, Chitosan, Poloxamer, Sericin	Superior ECM-like environment; enables minimally invasive delivery.	Low mechanical strength; rapid degradation limits long-term support.	rhBMP-2: 30 μg/scaffold	Mouse models; MSCs	Sulphated gelatine/rhBMP-2: Enhances receptor binding;	[79–84]
				PDGF-BB: 4.3 μg/mL	Mouse models; MSCs	Sericin/PDGF-BB: Stimulates osteogenesis	
				VEGF: 0.3 μg/mL; BMP-2: 0.3 μg/mL	DPSCs	Chitosan/VEGF and BMP-2: Synergistic bone repair;	
				–	MSCs	HS-collagen: MSC functions with minimal GFs.	
Rigid inorganic scaffolds	HAP, BCP, TCP, BG, CNTs	High load-bearing capacity; mimics natural bone mineral phase.	High fracture risk; limited intrinsic osteoinductive capacity.	BMP-7: 20 μg/mL	HBPCs	CHA/BMP-7: Enhances scaffold osteoconductivity;	[85–87]
				rhBMP-9: 100 ng/mL	ST2 cells	I-BCP/rhBMP-9: Synergistic bone formation;	
				–	MC3T3-E1; Rabbit models	TCP/PLGA: Facilitates long-term defect repair.	
Composite Interface Strategies	PLA, PEG, PCL, PLGA, PHA	Highly customizable degradation/mechanics; easy to process into complex shapes.	Hydrophobic surface hinders cell adhesion; acidic byproducts may cause inflammation.	–	Rabbit models	PEG-Gelatine/ BMP-2: Sustained GF release;	[88–91]
				bFGF: 5 μg/mL; BMP-2: 300 μg/mL	Dog models	DBM-Collagen/GFs: Accelerates tissue maturation;	
				–	Rat models	PLGA/MSCs: Cell-based bone regeneration;	
				VEGF: 2 μg/mL	MSCs; MC3T3-E1; Rat models	PP-GelMa/VEGF: Synergistic bone regeneration.	
Microcarrier Systems	MSNs, liposomes, Microspheres	High drug loading efficiency; precise control over release kinetics.	Difficult to scale/reproduce; risk of carrier migration from defect sites.	BMP-2: 1.5 mg/mL	Rat models	BCP-Collagen Microspheres/BMP-2: Sustained GF release;	[92–95]
				–	RAW 264.7; MSCs	Cu-MSNs: Modulates GFs for regeneration;	
				VEGF: 5 μg/mL	MC3T3-E1; Sheep models	SiHA/VEGF: Promotes coupled bone and vascular formation.	

*Note:* BG, Bioglass; CHA, Collagen-hydroxyapatite; PP, pearl powder; GelMa, fish-derived gelatine methacrylate; HBPCs, human boneprogenitor cells; SiHA, crystalline and nanocrystalline silicon-substituted hydroxyapatite.

### 3.1. Modulating early release kinetics *via* hydrated networks

The physical environment plays a key regulatory role in cell differentiation, migration and multiple behavioural manifestations. Hydrogels, which simulate natural osteomechanical characteristics, can effectively promote bone healing. Therefore, the mechanical integrity of the hydrogel-based delivery systems is crucial to achieve the effect of bone regeneration treatment [96]. In recent years, hydrogel materials have aroused a strong interest among scientists in multidisciplinary fields. This kind of material is composed of cross-linked hydrophilic polymers, which has the ability to store a large amount of water and can simulate the mechanical properties of human tissues through design, so it is especially suitable for BTE. Researchers have used natural and synthetic polymer-based hydrogels to deliver a variety of biologically active substances, such as GFs [97,98].

Injectable hydrogels are increasingly regarded as an effective carrier of cells in the fields of BTE and regenerative medicine. Their high water content and similar characteristics to the ECM make their ideal for enhancing cell culture and delivery mechanisms. Furthermore, they can be delivered to specific areas *via* minimally invasive techniques, adapting to irregular defects [99]. Researchers including Liu et al. [79] have created a catechol-chitosan hydrogel enhanced with zeolitic imidazolate framework-8 (ZIF-8) nanoparticles, which demonstrates remarkable adhesion, mechanical durability and antibacterial features. This hydrogel encourages rat bone marrow stem cells to produce VEGF, aiding in revascularization, while also releasing Zn^2+^ to enhance the expression of osteogenic markers, thereby significantly promoting vascularization, bone formation and healing in areas of bone defects.

While hydrogels are easy to inject, a critical parameter that must be monitored during translation is whether their degradation products cause local pH changes *in vivo* that could impair GF activity [100]. Furthermore, the clinical translation of the technology is restrained by the poor mechanical strength which limits its load-bearing applications [101], as well as the lack of *in vivo* evaluation in complexities fracture models rather than cranial/tibial defects. Additional validation is necessary for safety aspects, namely controlled GF release and long-term [102]. Future research should aim to create high-performance, smart-responsive hydrogels, such as those based on nanocomposites, and explore their potential in treating fractures and regulating the immune microenvironment to foster advancements in bone regeneration.

### 3.2. Achieving long-term GFs retention through structural rigidity

Although hydrogels are effective for initial release, late-stage bone remodelling necessitates a more sustained presence of GFs. Rigid inorganic scaffolds, such as hydroxyapatite (HAP), biphasic calcium phosphate (BCP) [103], TCP [104], bioactive glass (BG) [105] and carbon nanotubes (CNTs) [106], provide not only the essential mechanical strength for load-bearing defects but also serve as long-term GFs depots. Unlike soft tissues that rely on rapid diffusion, such bioabsorbable materials capture GFs through their rigid microstructures, or adsorb them in large quantities on the surface. As the material is gradually replaced by natural bone tissue, biologically active molecules will be gradually released [107]. This slow-release kinetic characteristics are highly consistent with the natural bone metabolism process.

For example, the structure and surface characteristics of BCP make it efficient in long-term delivery. Fujioka-Kobayashi et al. [86] confirmed that injectable BCP can strongly adsorb and slowly release rhBMP-9. This continuous retention significantly enhances the bone induction ability of the stent, resulting in a significant increase in the activity of alkaline phosphatase and the expression of osteoblasting markers (ALP and cyanate) in mouse bone marrow stromal (ST2) cells, while slightly reducing the initial cell adhesion. In addition, its injectable properties help fill complex defects while maintaining this controllable release characteristics [108]. Similarly, β-TCP is widely used to maintain ossogenesis and angiogenic signalling through a complete regeneration cycle [109]. In the sheep lumbar fusion model, Gadomski et al. [110] confirmed that the combined application effect of rhPDGF-BB and collagen β-TCP is comparable to that of autologous bone transplantation, providing a feasible alternative to human spinal fusion surgery.

However, matching the degradation rate of these rigid scaffolds with new bone formation remains a major translational challenge [111]. If the degradation rate of the stent is too slow, it will physically hinder the growth of the tissue. If the degradation is too fast, there is a risk of premature release of drug doses. In addition, long-term implant safety and the lack of standardized large animal studies to accurately evaluate the systemic effect and human predictability are still major obstacles that must be solved in order to achieve a wider range of clinical applications [112].

### 3.3. Overcoming kinetic limitations *via* composite interface strategies

Inorganic synthetic materials are often used as bone scaffolds because of their significant compatibility with biological tissues, which is conducive to the proliferation and adhesion of bone cells. However, its brittleness may damage bone integrity, and it is usually necessary to add appropriate additives to enhance its performance [113,114]. In the field of BTE, natural polymers such as collagen, gelatine, alginate, chitosan and hyaluronic acid, as well as synthetic materials such as Poly (lactic-co-glycolicacid) (PLGA), Polyethylence Glycol (PEG), Polycaprolactone (PCL), Polyhydroxyalkanoates (PHA) and Polylactic acid (PLA) are often used as additives [115,116].

By combining inorganic materials with polymers, composite supports can significantly improve mechanical properties and realize the controlled release of biologically active factors. Murali et al. [117] developed an affinity-selected heparan sulphate (HS), which effectively heals rabbit bone defects by enhancing endogenous BMP-2 activity. Furthermore, its integration with β-TCP implants significantly improves structural characteristics and promotes bone formation [118]. In addition, multi-layer designs can simulate natural gradient structures, promoting synchronous bone and cartilage regeneration. Huang et al. [119] have developed a freeze-dried sponge impregnated with TGF-β3, recombinant human collagen and chitosan. This innovative porous structure significantly improves the expression level of Runt-related transcription factor 2, BMP-2 and type I collagen, which are crucial for the repair of skull defects.

Composite scaffolds combining inorganic materials with natural or synthetic polymers offer enhanced mechanical properties and controlled release of bioactive factors for bone and osteochondral regeneration. However, clinical translation requires further validation of long-term biodegradation profiles, immunogenic responses to polymer components [120], and standardized manufacturing to ensure scaffold consistency and safety in load-bearing applications [121].

### 3.4. Microcarrier systems and sequential delivery

Bone scaffolds made from microspheres allow for prolonged drug release using absorbable microcarriers that apply GFs over specific periods of time. Microspheres are range from 1 to 1000 μm and are mainly formulations composed of GFs or bioactive substance dispersed in or adsorbed onto a polymeric matrix. Because of their extensive size and volume, they are the ideal materials to carry GFs and allow their loading and release [122,123].

The customized release of these therapeutic agents can be achieved more effectively through the integration of various GFs into the delivery matrix or particle carriers [124,125]. Microgel bone regeneration units containing demineralized bone matrix and VEGF were developed through photopolymerization and microfluidic techniques by Hao et al. [126]. This system precisely regulates the microenvironment to induce BMSC osteogenic differentiation, so as to successfully repair tibial defects in rabbits with both morphological and functional recovery. Likewise, Erfan et al. [127] prepared uniform PLGA microcarriers encapsulating VEGF at its interior core with microfluidic techniques. The surface was modified with polydopamine that was loaded with BMP-2 and released over time. These microcarriers facilitated the proliferation of MSCs and their osteogenic differentiation. When combined with endothelial cells in an injectable hydrogel, they effectively stimulated vascularized bone formation in rat models.

Mesoporous Silica Nanoparticles (MSNs) have emerged as the carrier of choice for bioactive molecule delivery in BTE and cartilage tissue engineering owing to their excellent physicochemical stability, biocompatibility, large surface area and tuneable pore structure, and easy functionalization [128,129]. The controlled loading and release of therapeutic agents makes them different from common polymer-based systems [130]. Shen et al. [131] took the lead in developing MSNs loaded with FGF-2, which can promote ossification differentiation through the Wnt/β-catenin signalling pathway and significantly improve the bone healing ability of the femoral model. In addition, when combined with BMP-2, MSNs integrated stents showed remarkable therapeutic effect in cartilage regeneration and pulp regeneration of the periapical inflammation model [132,133].

Microsphere systems and MSNs were developed for the controlled delivery of osteogenic and angiogenic factors for bone regeneration. Studies show that they can induce stem cell differentiation and repair critical-sized defects *in vivo* by the designed release mechanism. The employed carriers must be assessed for safety with respect to examining whether it will be degradable and its degradation rate; verifying the immunogenicity of the silicon-based material used [134]; analyzing the biocompatibility of its degradation by-products within the expected time frame; and evaluating its ability to control the release of GFs in time and space to avoid off-target effects [135]. Standardized manufacturing, sterilization validation and preclinical models to assure compliance with regulation are key to reproducible safety profiles prior to human use.

## 4. Advanced synergistic and responsive delivery strategies

In order to meet the needs of controlled release of GFs, scientists have invested a lot of resources to upgrade the basic delivery system to a precision platform to realize the on-demand release of GFs. The main objectives of developing these advanced delivery systems include enhancing the ability to control the release of GFs and improving the therapeutic effect by simultaneously delivering multiple GFs [136]. Unlike the static platforms discussed in Section 3, these strategies transform scaffolds from passive carriers into dynamic interfaces that respond to biological or physical cues (Table 3). This chapter focusses on how to realize the space-time control, environmental response and multi-factor synergy of GFs through advanced delivery platforms, and evaluates the translational potential of these innovations, focusing on the trade-off between bio-sophistication and clinical feasibility.

**Table 3.** Summary of strategies, methods and effects of multi-modal synergistic GFs in promoting bone regeneration.

Synergistic strategy	Effect	Case studies	Intervention/dose (unit)	Model type/species	Endpoints	References
Multi-factor time-release	Mimics natural healing stages to synchronize angiogenesis and osteogenesis.	Nanofibres scaffold	BMP-2: 379 ± 34 μg/mL; VEGF: 403 ± 7 μg/mL	MSCs	Achieves 21-day differentiation;	[137]
		HMSN@pDA microspheres	PDGF-BB: 100 ng/mL; IGF-1: 100 ng/mL; TGF-β3: 100 ng/mL	MSCs; rabbit models	4-week cartilage regeneration;	[138]
		Alginate and PEGDA	BMP-2: 500 ng/mL; VEGF: 500 ng/mL	MC3T3-E1 cells	Sustained 14-day dual delivery;	[139]
		nBGs	VEGF: 2 μg/mL	HUVEC	Efficient immobilization (>80%).	[140]
Genetically engineered cells	Provides stable GF expression with localized structural support.	3D fibrin scaffold +BMSCs	–	Rat models	Enhances calvarial regeneration;	[141]
		GelMA microspheres +BMSCs	BMP-2: 200 ng/mL	Rabbit models	80–90% release in 5 days;	[142]
		BCP+BMSCs	–	MSCs;HUVEC; Mouse models	Synergistically increases osteo/angiogenesis.	[143]
Bionic ECM signalling network	Actively guides cell behaviour and GF kinetics *via* native-like physical support.	FG/Fn/Hep hydrogel	BMP-2: 125 ng/mL	MC3T3-E1 cells; Rat models	Sustained 12-day BMP-2 release;	[144]
		U-PA/Plg system	–	HSPCs; MSCs; Mouse models	recruits macrophages for repair;	[145]
		BioCaP-sericin scaffold	TGF-β3: 1 μg/mL; BMP-2: 1 μg/mL	osteoclast; Rabbit models	66% sustained release over 19 days.	[146]
Stimulus-responsive release	Enables precise on-demand release triggered by internal or external factors.	Liposome-nanoparticles	rhBMP-2: 10 μg/mL	Mouse models	Ultrasound-triggered induction over 28 days;	[147]
		IF-8-PDA scaffold	PDGF: 10 μg/scaffold	MSCs; Rat models	It releases PDGF(43.28 ± 2.45% over 28 h);	[148]
		P407-GelMA hydrogel	IGF-1: 100 ng/mL	MSCs; Rat models	Programmed peak release on Day 13;	[149]
		Alkaline shear-thinning hydrogel	TGF-β1: 0.7 ng/mL	Rat models	Promotes osteogenic differentiation.	[150]
Electrospinning	Biomimetic porosity for mechanical support and vascular-assisted growth.	Nanofibre scaffolds	VEGF: 2.5 μg/mL BMP-2: 2.5 μg/mL	MSCs	Initial burst (Day 1) +sustained release (80% at 14d).	[151]
3D Printing		3D-printed PCL	TGF-β1: 20 ng/mL	MG-63 cells	35-day sustained release;	[152]
		3D-printed PLLA/PCL-IPMs	VEGF: 500 ng/mL	Rat models	It synergistically promotes osteogenesis/angiogenesis.	[153]
4D Printing	Using smart materials that self-transform over time	4D-printed Fe3O4- PLLA scaffolds	–	Raw 264.7; MSCs; Rat models	Induces M2 macrophage polarization for regeneration.	[154]
5D Printing	High-res precision printing of curved/concave layers with 3–5× strength of standard 3D prints	5D Printing Gel/GelMA	TGF-β1: 200 ng/mL	MSCs	Most cells survive the printing process.	[155]
6D Printing	Able to alter its shape or properties in response to environmental stimuli.	–	–	–	Integrating 4D responsiveness with 5D complex architectures.	[156]

*Note:* PEGDA, polyethylene glycol diacrylate; FG/Fn/Hep, fibrin glue/fibronectin/heparin; nBGs, nanosized bioactive glass particles; u-PA, urokinase-type plasminogen activator; Plg, plasminogen; BioCaP, bio-inspired calcium phosphate coating; PDA, polydopamine; IPMs, injection-moulded microchannel; PLLA, nanofibrous poly (l-lactide); HSPCs, haematopoietic stem and progenitor cells; MG-63, human osteosarcoma cells.

### 4.1. Multifactor synergy and temporal control

The controlled release of GFs according to time and space is important for the success of combination strategies. The spatiotemporal control of factor combinations can be achieved by using controlled-release systems. GFs exhibit distinct, stage-specific requirements during the regenerative process. In chondrogenesis, TGF-β3 and FGF-2 demonstrate a clear division of labour. *In vitro* studies indicate that TGF-β3 induces early changes in chondrocyte morphology and ECM secretion, while FGF-2 acts as a primary regulator of cell proliferation [157].

**Figure 4.**
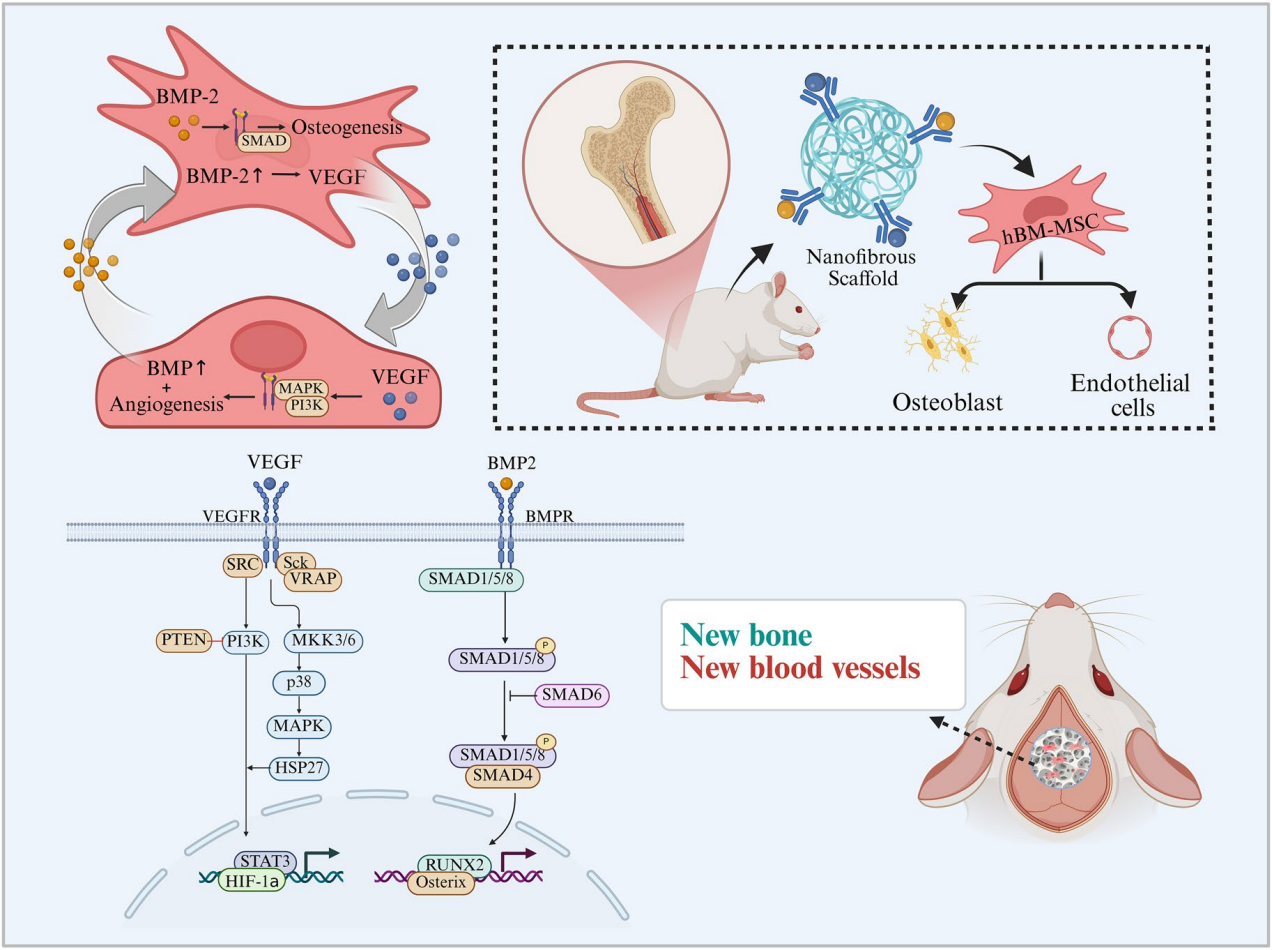
Synergistic signalling pathways and feedback loops of VEGF and BMP-2. This diagram details the molecular mechanisms underlying the angiogenic-osteogenic coupling: VEGF activates MAPK/PI3K pathways in endothelial cells while triggering paracrine BMP-2 secretion. In turn, BMP-2 stimulates MSC osteogenesis *via* SMAD signalling and upregulates VEGF, establishing a reciprocal positive feedback loop. This molecular synchronization ensures coordinated microvascular maturation and bone matrix mineralization, providing a mechanistic basis for their combined clinical efficacy.

Furthermore, utilizing PLGA microparticles to regulate the release kinetics of BMP-2 and FGF-2 has been shown to modulate collagen deposition and the expression of osteogenic/chondrogenic genes, providing a robust model for optimizing regenerative therapies [158]. In Bone Regeneration, the synergy between angiogenesis and osteogenesis is a primary research focus (Figure 4). The combined delivery of VEGF and BMPs can act on both endothelial cells and osteoblasts. For example, increased BMP expression at the fracture site can promote osteogenesis and stimulate angiogenesis at the same time [159].

In order to overcome the limitation of insufficient stability of exogenous proteins, research has turned to a platform that can achieve continuous endogenous expression. Yao et al. [160] developed a decellular scaffold system that anchors the engineered exocrine loaded with VEGF on the 3D printed porous scaffold through CP05 peptide. The system significantly enhances the differentiation ability of mesenchymal stem cells into osteoblasts and promotes vascular remodelling *in vivo*. Geng et al. [161] used Modified mRNA (modRNA) technology to genetically modify BMSCs to express BMP-2 and VEGF-A, showing superior healing compared to traditional protein delivery. From a translational perspective, these living factories provide a more stable therapeutic window than single-dose injections.

While multi-factor synergy significantly outperforms monotherapy in preclinical models, the complexity of dual-release kinetics increases regulatory hurdles. Clinically, the more factors involved, the higher the risk of off-target effects and the more difficult it is to define a standard dose for heterogeneous patient populations [162]. When promoting clinical transformation, safety measures need to consider the following factors: the immunogenicity of viral or non-viral vectors [163], the off-target effect associated with gene therapy [164], the ectopic tissue formation that may be caused by the out-of-control release of factors, and the long-term stability of genetically engineered cells or exocrine bodies *in vivo*.

### 4.2. Spatial control and microenvironment construction

Compared with traditional materials engineering, GFs administration through the cell carrier integrated into the delivery system marks a paradigm shift to a living precision platform. These systems use stem cells that can naturally secrete GFs or achieve targeted production through genetic modification [160,165]. Cell-based treatment techniques mainly use the strong regeneration ability of stem cells to promote their growth and differentiation at the injured site, while enhancing the activation and differentiation of autologous stem cells in the body, so as to assist in repairing bone defects [166].

Biofunctional stents provide the necessary spatial guidance for the utilization of the regeneration potential of BMSCs, which is of key clinical significance for large-scale bone tissue generation [167]. Chen et al. [141] show that three-dimensional fibrin stents containing BMSCs and concentrated PDGF can promote the continuous release of VEGF. In the mouse model, this promotes angiogenesis and centripetal bone regeneration. Further progress includes releasing hydrogels of nitric oxide (NO), which enhance bone-vascular coupling by activating the NO/cGMP signalling pathway and regulating the parasecretory activity of BMSC [168].

A key advancement in spatial control is the transition from static support to dynamic microenvironments. It allows clinicians to trigger release only at the defect site, minimizing systemic exposure. Casanova et al. [169] designed a fibrin-gold nanoparticle hydrogel that responds to near-infrared light. The system can realize the on-demand light and heat release of BMP-2, which significantly enhances the generation of mineralized tissue in the skull defect model. This shows how the engineering bracket can change from static support to dynamic interface, thus determining the delivery of BMSC behaviour and GF in time and space.

The primary bottleneck for cell-based spatial control is the tumorigenic risk and immunogenicity of modified cells [170]. The scalability of such living platforms is limited by the high cost of cell expansion and the stringent requirements for maintaining cell viability during surgical implantation. The most important thing is that the secretory kinetics of GFs must be precisely regulated. If the release dose cannot be strictly controlled, it is very easy to cause systemic adverse reactions or ectopic tissue formation [171]. The risk of this dynamic disorder is particularly prominent in highly bioactive factors such as BMP-2, which has become the main bottleneck in promoting the transformation of these technologies into clinical applications.

### 4.3. Harnessing bionic ECM signalling networks

ECM consists of various biomolecules like glycoproteins, glycosaminoglycans (GAGs) and Proteoglycans, produced by local cells [172]. At first the ECM was considered just a natural backdrop maintaining the structure of the different tissues. It is important in several cellular processes such as migration, cellular growth and metabolic processes [173]. Due to the binding sites of various cytokines and GFs, it dictates how they act on cells. ECM plays a crucial role in regulating the activities of GFs in life [174].

The use of ECM to control GF signal conduction and space-time release has promoted the development of a variety of biomaterial delivery systems for bone regeneration [175]. These systems integrate ECM components that simulate the function of natural organisms. Although adhesion-promoting proteins such as fibrin, layered adhesion protein and type I collagen help cells adhere and activate key intracellular signalling pathways [176], GAGs such as HS provide GF binding and stability. Chemical and continuous release function [177]. Notable advancements include the work of Julius et al. [178] who developed a streptavidin platform for co-presentation of HS, BMP-2 and the integrin ligand cRGD exhibiting synergistic enhancement of osteogenic differentiation. Strategies parallel to this involve the integration of ECM-derived functional elements, as exemplified by scaffolds that couple BMP-2-functionalized extracellular vesicles to antimicrobial chitosan-aloe vera matrices to promote osteogenesis and hinder bacterial biofilm formation simultaneously [179]. ECM-inspired designs, as demonstrated by these diverse methodologies, have the ability to generate multifunctional, regenerative environments.

The use of ECM in biological materials can also achieve superior control of GF signal conduction and space-time release, thus promoting bone regeneration. The system composed of ECM components such as fibre-connecting protein and HS can promote cell adhesion and continuous delivery of GFs. For example, the system with both HS and BMP-2 induces bone formation in a collaborative way. In clinical transformation, safety considerations must solve the following problems: immunogenicity of ECM-derived ingredients [180], inter-batch variability of natural extracts, potential unexpected GF interactions [181], and long-term biocompatibility of composite stents in load-bearing applications.

### 4.4. Stimulus-responsive release

The stimulus-responsive drug delivery system (SRDDS) marks a paradigm shift from passive diffusion to active targeted delivery on demand. By responding to specific external stimuli or internal microenvironmental changes, these systems can prevent the premature release of drugs, thus minimizing systemic toxicity and significantly improving the treatment efficiency of the target site [182,183].

External stimulation enables clinicians to non-invasively regulate the release kinetics of GF with high time accuracy. Electric fields have been shown to promote the proliferation and differentiation of MSCs. This mechanism involves the upregulation of endogenous BMPs and the activation of calcium-calcion and TGF-β pathways, which together drive significant new bone formation [184]. The lipid-rhBMP-2 nanocomposite developed by the Gazelle et al. [147] can achieve the controlled release of GFs through non-thermal ultrasonic irradiation. *In vivo* experiments show that local bone formation occurs strictly after irradiation, and its release rate is positively correlated with ultrasonic pressure and irradiation time.

Researchers are increasingly paying attention to hydrogels and micro-nanomaterials that respond to endogenous signals, such as enzymes, pH or redox gradients. Lee et al. [185] designed a double-layer freezing gel system to realize the sequential release of VEGF and BMP-4. The outer layer provides fast angiogenesis signals, while the inner layer ensures the gradual release of osteogenic factors, which significantly promotes *in vitro* differentiation and regeneration of skull defects. Yuan et al. [186] developed a responsive hydrogel composed of chitin liquid crystal and mesoporous bioactive glass. The system can specifically respond to the polarized microenvironment of M2 macrophages to release BMP-4 and achieve immunomodulation through Ca^2+^/Si^4+^ ions, thus promoting the bone repair of the skull defect model.

Although SRDDS shows high accuracy in preclinical models, the application of these intelligent materials to clinical practice still faces major analytical and safety challenges. To get the clinical translation aspect right, critical safety issues regarding responsive nanomaterials concerns their biocompatibility and clearance [187]. In addition, the long-term stability of the off-target reaction and trigger mechanism of external stimuli should also be considered. Finally, it is also necessary to verify the dose control that is difficult to prevent ectoplastic tissue formation or systemic toxicity [188].

### 4.5. Advanced fabrication technologies: Bridging design and precision delivery

In order to transform complex design principles such as physical microenvironment modelling and multi-layer composite strategies into functional implants, 3D printing and electrostatic spinning technology have become indispensable engineering tools. These platforms can realize the precise spatial arrangement of materials and GFs, thus promoting the reconstruction of complex bone microenvironments [189,190]^.^ The latest research shows that scaffolds made from 3D-printed bone-cartilage nanocomposites, which incorporate functional oil-in-water emulsions, can greatly enhance cellular growth in adjacent bone areas. Another method is to combine a 3D printed porous hollow cage-like stent with rhBMP-2 and MSCs for bone transplantation [191]. Additionally, Du et al. [192] used 3D-printed water-soluble polyvinyl alcohol sacrificial moulds to create polymerized polyhydroxyethyl acrylate scaffolds. This innovative structure notably enhanced cell infiltration, proliferation and osteogenic differentiation, while also allowing for efficient BMP-2 loading and controlled release, thereby improving vascularization and bone regeneration in mouse cranial defects.

**Figure 5.**
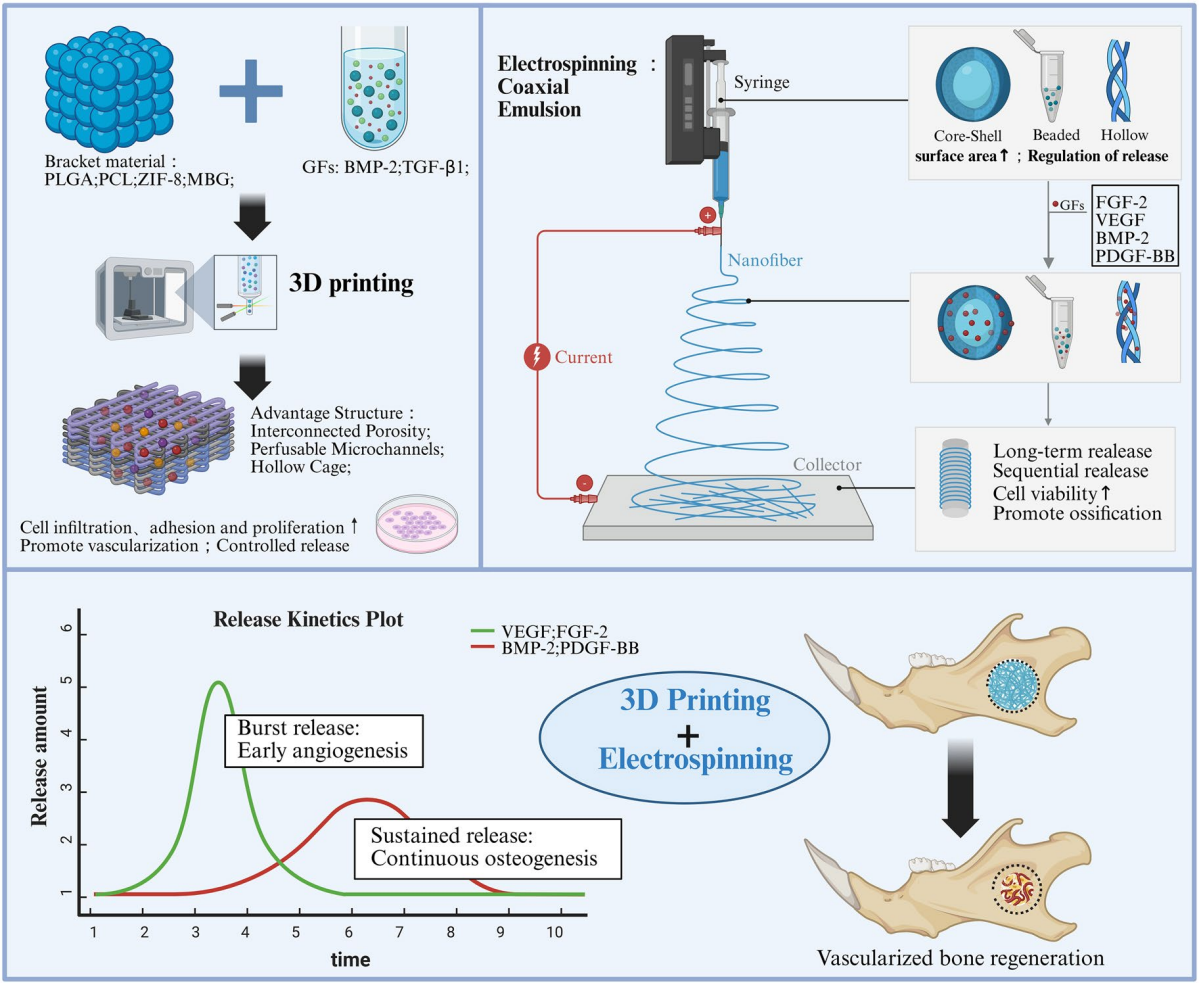
Synergistic integration of 3D printing and electrospinning for vascularized bone regeneration. 3D Printing (left): Creates macro-porous scaffolds with microchannels for structural support and spatial cell guidance; Electrospinning (right): Develops ECM-mimetic micro-nano fibres optimized for programmed bioactive factor release; Functional Integration: Sequential release kinetics (early angiogenic burst followed by sustained osteogenesis) drive functional *in vivo* bone repair.

Nanofibrous scaffolds produced *via* electrospinning are available in a variety of morphologies such as hollow, core-sheath and beaded structures that can be engineered by altering processing parameters. The biomimetic surface roughness and mechanical properties, as well as the multilayer architecture, fibres mimic the native extracellular microenvironment and functionality of bone to enhance cell adhesion and osteogenic behaviour [193,194]. These scaffolds also serve as sustained delivery platforms for osteogenic GFs. Similarly, PDGF-BB incorporation by emulsion electrospinning upregulates the osteogenic markers according to Briggs et al. [195]. The study which involves encapsulation of FGF-2 in coaxial electrospun fibres is found to enhance the viability of fibroblasts [196]. These findings may suggest the successful potential of nanofibrous systems. Together, these strategies demonstrate how electrospun matrices designed in a structural and biochemical way can spatially and temporally direct cellular behaviour to enhance bone regeneration (Figure 5).

In order to meet the clinical demand for dynamic responsiveness and mechanical optimization, manufacturing technology has undergone a dimensional evolution, among which low-temperature 3D and 4D bioprinting technologies are specially used to maintain the biological activity of heat-sensitive GFs [197,198]. Traditional 3D printing meets the macrostructural needs of cell infiltration by providing personalized geometric matching and porous frames, while 4D printing further expands these functions by integrating time dimensions. This enables the stent to respond to biological stimuli and cause preset morphological or functional transformations, thus solving the limitation that static implants cannot adapt to the evolution of the repair stage [199,200]. In addition, 5D printing has overcome the limitations of standard printers in replicating complex anatomical curvature by enhancing mechanical integration and stress distribution by using a five-axis system, which has been verified in the case of gene activation stents to coordinate sequential blood vessel priority regeneration [201,202]. 6D printing technology finally realizes the perfect integration of 4D response intelligence and 5D structural advantages, and manufactures intelligent implants through computing optimization technology [156], such implants maintain structural integrity in load-bearing applications and can perform complex biological functions under physiological pressure.

These platforms are increasingly sophisticated, their clinical application path is still subject to many engineering and biological challenges. Although 5D and 6D printing technologies have extremely high accuracy, their prohibitive equipment costs and extremely stringent standards for printing parameters make cross-laboratory reproducibility and large-scale industrial production the most significant current bottlenecks [203]. In addition, the physical or chemical conditions involved in many high-resolution technologies may impair the biological activity of GFs. The current preclinical research still mainly relies on secondary treatment processes such as surface spraying or hydrogel sealing, which may lead to insufficient binding affinity or unpredictable release dynamics [204]. Clinical transformation must give priority to long-term biocompatibility and controllable biodegradability. It is urgent to carry out safety research on the local release of bioactivators at the target site to prevent off-target effects and adverse immune reactions [205].

## 5. Translational barriers and safety challenges

To provide a clear roadmap for clinical translation, we conducted a cross-sectional evaluation of both the fundamental biomaterial platforms and the advanced synergistic strategies, which focuses on five critical dimensions: safety, mechanical properties, sustained release kinetics, cost-effectiveness and translational risk. This comparison highlights the trade-offs between biological precision and practical clinical implementation (Table 4).

**Table 4.** Critical comparison of foundational platforms and collaborative innovation strategies.

Category	Strategy	Safety & biocompatibility	Mechanical properties	Sustained release kinetics	Cost-effectiveness	Translational risk	References
Foundational Platforms	Hydrogels	Biomimetic and non-toxic	Poor structural integrity	High burst release	Low-cost fabrication	High regulatory maturity	[206,207]
	Inorganic Scaffolds	Potential for brittle debris	High compressive strength	Long-term retention	Standardized production	Degradation matching issues	[208,209]
	Polymer Scaffolds	Highly predictable behaviour	Tailorable stiffness	Enables multi-stage release	Scalable manufacturing	Well-established safety profiles	[210,211]
	Micro-nano Carriers	Protects labile payloads	Integrated into matrices	Linear release	Complex encapsulation	Batch consistency challenges	[212,213]
Advanced Strategies	Multifactor Control	Complex signalling	Depends on host material	Mimics healing cascades	High research and development costs	Complex interactions	[214,215]
	Spatial Control	Targeted localized delivery	Integrated structural design	Localized behaviour	Precision-dependent	Manufacturing complexity	[216,217]
	Bionic ECM	High biological fidelity	Matrix-dependent	Ultra-slow release	Complex bio-functionalization	Stability and safety concerns	[218,219]
	Stimulus-responsive	Potential trigger toxicity	Stimuli-dependent	Zero premature release	Sophisticated chemistry	Off-target and hardware risks	[220,221]
	Advanced Fabrication Technologies	Low residual stress	Anisotropic architecture	Synchronizes bone/vessel growth.	Expensive equipment	Regulatory hurdles for combination products	[222,223]

*Note:* PEGDA, polyethylene glycol diacrylate; FG/Fn/Hep, fibrin glue/fibronectin/heparin; nBGs, nanosized bioactive glass particles; u-PA, urokinase-type plasminogen activator; Plg, plasminogen; BioCaP, bio-inspired calcium phosphate coating; PDA, polydopamine; IPMs, injection-moulded microchannel; PLLA, nanofibrous poly (l-lactide); HSPCs, haematopoietic stem and progenitor cells; MG-63, human osteosarcoma cells.

A major challenge in the application of GFs in BTE is the occurrence of ectopic bone formation [224]. The current delivery method usually leads to leakage or off-target release of GFs, which can induce local inflammation or immune response [225]. In addition, existing delivery systems show insufficient targeting capacity, and their disfunction at low doses requires a larger amount to achieve the desired effect, thus increasing the risk of off-targeting effects [226].

Furthermore, the long-term biological significance of GFs exposure is still controversial. Many studies ignore a comprehensive assessment of tissue remodelling, immune response and metabolic changes after long-term GFs delivery-reflecting a broader process of immune metabolism reprogramming [227]. In addition, GFs may affect distal organs or systems through systemic effects, which have caused particular concern among individuals with a history of cancer through the interaction between parasecretory signals and the tumour microenvironment [228].

Another critical challenge lies in controlling the dose-reaction relationship is a major challenge [229]. Due to the rapid degradation of enzymes in the body, the usual delivery method requires superphysiological doses. The mismatch between the degradation rate of the carrier and the release kinetics of GFs is also common [230]. The main problem of the complex gene delivery system is the inability to accurately control the time and intensity of transgenic expression and the therapeutic activity [231].

Moreover, the lack of a standardized scheme for the long-term safety evaluation of GFs delivery system hinders clinical transformation [232]. The high cost of GFs and the complexity of mixed hydrogel or microcarriers constitute substantial obstacles to mass production. There are still problems in achieving consistency and stable quality control between batches, especially for natural polymer scaffolds, such as collagen [233] and gelatine, in which the variability of source and purification affects uniformity. Manufacturing must also meet the requirements of strict good manufacturing standards, especially for systems designed for continuous GFs release, which requires precise control of component proportions and spatial organization. At present, most delivery systems are still limited to laboratory-scale research, because large-scale production to ensure stable performance is the main obstacle to industrial and clinical use.

## 6. Conclusions and perspectives

This review focusses on the synergistic enhancement of GF delivery system in bone repair. Although platforms such as hydrogel, inorganic carriers and polymer carriers have successfully improved the stability of GFs, composite scaffolds and ECM inspiration systems currently represent the most clinically realistic strategies. They are released through the effective balance of mechanical support and space-time control of bioactive factors. However, the key unresolved challenges are still hindering a wide range of clinical transformation. These challenges include the risk of ectopic bone formation due to inaccurate dose control, the lack of standardized programs for long-term safety assessment, and the engineering complexity and high cost that hinder large-scale production. In the future, the combination of 3D bioprinting with vascularization technology and artificial intelligence is crucial to establish clinical standards and transform GF printing technology into bedside treatment.

## Data Availability

No data were generated in this work. All data discussed in the manuscript are derived from previously published studies, which are cited in the reference list.
